# Case Report: presentation of pleomorphic liposarcoma arising in a borderline phyllodes tumor

**DOI:** 10.1016/j.ijscr.2018.10.038

**Published:** 2018-11-01

**Authors:** Yalçın Polat, Gözde Arslan, Abud Kebudi, Duygu Düşmez Apa

**Affiliations:** a University of Biruni, Faculty of Medicine, Department of Pathology, Protokol Yolu No: 45, 10. Yıl Cd., 34010 Zeytinburnu, İstanbul, Turkey; b University of Maltepe, Faculty of Medicine, Department of Radiology, University Medical Faculty, Department of Feyzullah Cad., No: 39 34843, Maltepe Istanbul, Turkey; c University of Okan, Faculty of Medicine, Department of General Surgery, Aydınlı Yolu Cad., Aydemir Sk. No: 2, 34947 İçmeler, Tuzla, İstanbul Turkey

**Keywords:** Case report, Breast, Histopathology, Liposarcoma, Philloides tumor

## Abstract

**Introduction:**

The stromal component of Phyllodes tumors represents the neoplastic portion of the lesion. A variety of associated malignancies can arise from PTs, with its dual population of cells.

**Presentation of case:**

We share a very rare case of “borderline”breast phillodes tumour with a 75% liposarcoma component. A 48-year-old woman presented at the surgical clinic with a mass in the left breast. On mammograms, the lesion was dense with radiolucent areas inside which were thought to be compatible with fat. The mass was assumed to be breast imaging-reporting and data system (BIRADS) 3 on sonography as it was well contoured. The needle biopsy was reported to be consistent with a fibroadenomatous lesion. The lesion was completely excised. On microscopic examination, the lesion has benign mammary duct epithelium lining the slit-like spaces. The stroma composed of fibrous and adipous tissue. The fibrous part was cellular, and several cells appeared bizarre. Adipous tissue replaced most of the stroma and was diagnosed as pleomorphic liposarcoma.

**Discussion:**

According to the 3 tiered grading criteria of PTs, our case normally suits in borderline category, without liposarcoma component. Heterologous sarcomatous elements usually accompany high grade PTs. Rare cases of benign and borderline PTs with sarcoma component have been reported.

**Conclusion:**

The presence of a malignant heterologous component places the tumour into the malignant category regardless of other histological features.

## Highlights

• A variety of associated malignancies can arise from PTs, with its dual population of cells.
• Heterologous sarcomatous elements usually accompany high grade PTs.
• The presence of a malignant heterologous component places the “Philloides tumor” into the malignant category regardless of other histological features.

## 1 Introduction

Phyllodes Tumors (PTs) are biphasic fibroepithelial lesions; with clefts lined with a bilayer of epithelium and myoepithelium within a cellular stroma. The stromal component, represents the neoplastic portion of the lesion. Like fibroadenomas, PTs originate from fibroblasts of the specialized periductal stroma [1,2].

The term “cystosarcoma phyllodes” was chosen to emphasize the leaf-like pattern and fleshy gross appearance of the lesion. PT is the currently preferred terminology. In the 4th edition of the WHO Classification of Tumours of the Breast, the neutral term 'benign fibroepithelial neoplasm” was favoured, where there is histological uncertainty in distinction from a benign phyllodes tumor [3].

Unlike fibroadenomas, PTs are potentially aggressive neoplasms that enlarge progressively, can invade adjacent structures, and sometimes recur after enucleation; and rarely metastasize. Benign PTs can be difficult to distinguish from fibroadenomas, while malignant PTs can grow in size quickly and metastasise early [2].

PTs occur over a wide age range. The median age of presentation is 45 years, 20 years later than that for fibroadenomas (FA). They are more prevalent in the Latin American and Asian populations. The reported average size of PTs is 4 to 5 cm, ranging from 1 cm to larger than 20 cm [1].

PTs are heterogenous in their gross appearance. A solid mass with cystic areas is demonstrated in most of the cases. Whorled appearance with curved clefts resembling leaf buds is seen in large tumors. Foci of necrosis, mucoid changes and hemorrhage may also be present [4]. The stroma is either slightly more cellular than fibroadenoma, or frankly sarcomatous [[4], [5], [6]].

Radiologic and histopathologic findings of PTs may be misleading. We report a rare case of breast phillodes tumour with a 75% liposarcoma component. The patient was managed in an academic hospital. This work has been reported in line with the SCARE criteria [7].

## 2 Case report

A 48-year-old woman presented at the surgical clinic with a mass in the left breast. The patient remarked that the mass had been present for two years. The patient had no history of nipple discharge or hormone treatment. There was no family history of breast cancer. There were no palpable left axillary lymph nodes, and laboratory tests revealed no significant findings.

### 2.1 Imaging

**Fig. 1:**
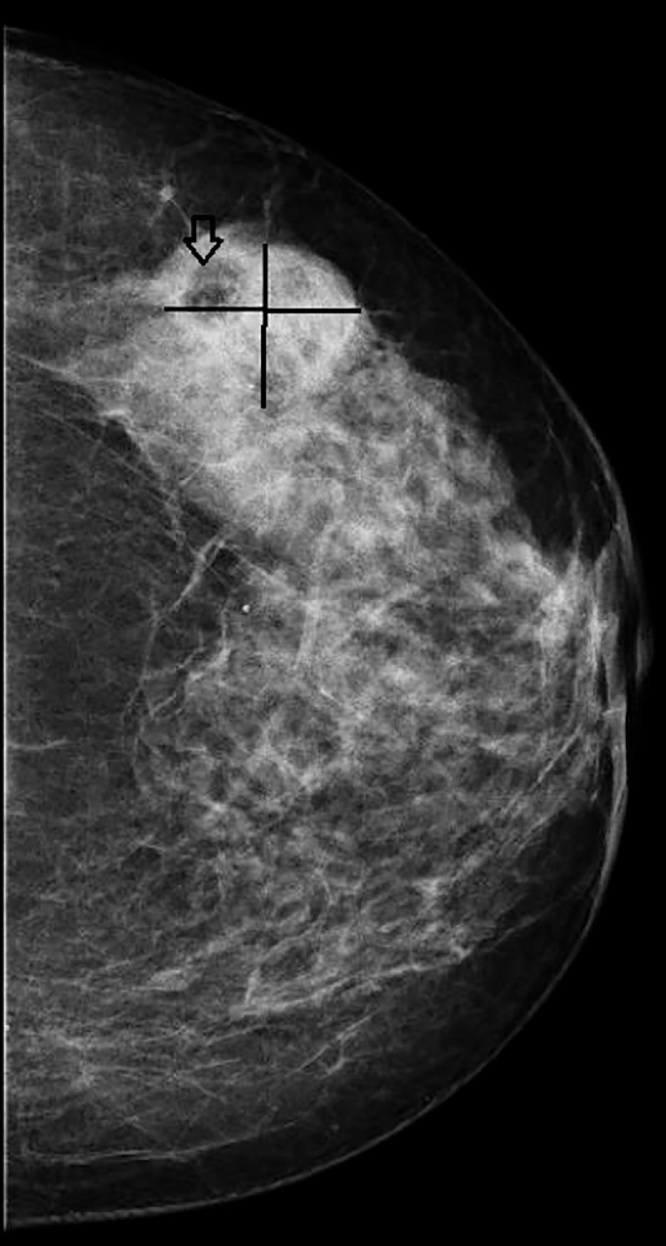
On mammograms the lesion was dense with radiolucent areas inside which were thought to be compatible with fat.

**Fig. 2:**
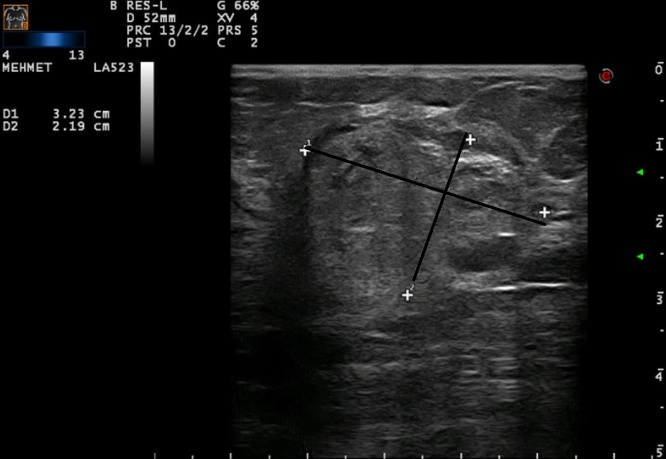
On sonographic imaging the lesion had smooth contours and was hypoechogenic with large hyperechoic components in between.

On mammograms the lesion was dense with radiolucent areas inside which were thought to be compatible with fat (Fig. 1). On sonographic imaging the lesion had smooth contours and was hypoechogenic with large hyperechoic components in between (Fig. 2). The mass was assumed to be breast imaging-reporting and data system (BIRADS) 3 on sonography as it was well contoured. A sonography guided tru cut biopsy was performed with a 16 Gauge needle.

### 2.2 Histopathology of tru cut biopsy

The sampling consisted of a fragmented tissue containing a few epithelial tubular formations in fibrous stroma with myxomatous degeneration. There were no atypical epithelial or stromal cells. A description of the lesion was made and was reported to be consistent with a fibroadenomatous lesion.

The lesion was completely excised.

### 2.3 Pathology of excisional biopsy

**Fig. 3:**
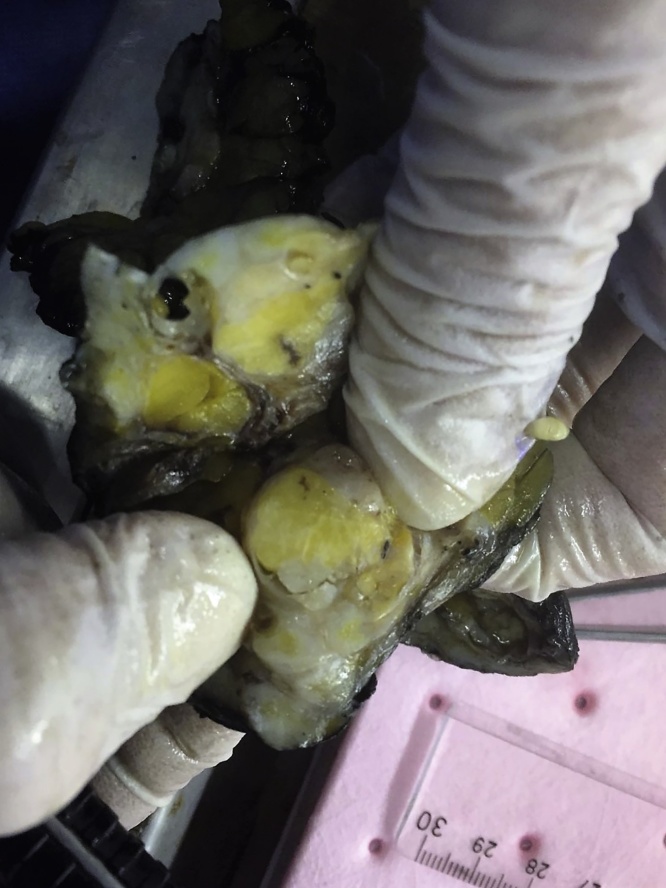
Cut surface of the material revealed, a yellow colored, elastic, firm mass with relatively well-defined lobulated contours.

The surgical specimen measured 60 × 50 × 40 mm, with an overlying skin measured 50 × 20 mms. Cut surface of the material revealed, a yellow colored, elastic, firm mass with relatively well-defined lobulated contours, measuring 27 × 25 × 15 mms. A free surgical margin of at least 3 mms was measured (Fig. 3).

**Fig. 4:**
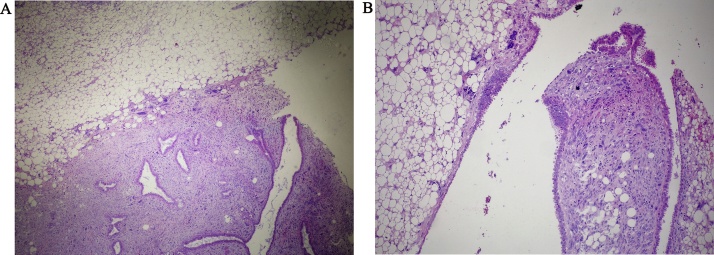
(a) The lesion is composed of two to three cell layers thick, benign mammary duct epithelium lining the slit-like spaces, and a cellular spindle cell stroma. (b) The cellular mesenchymal stromal elements protrude into cyst-like spaces in a leaf-like configuration.

**Fig. 5:**
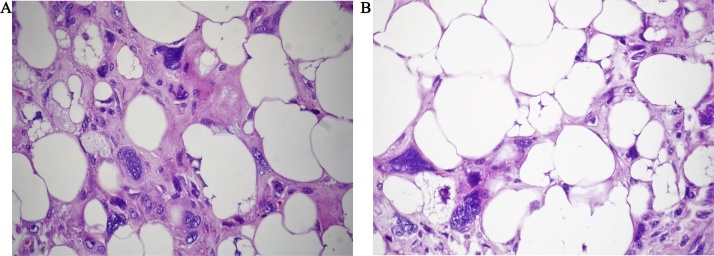
(a) Adipose tissue contained a significant number of bizarre cells; with a large cytoplasm and a lobulated nucleus. (b): These cells were evaluated as “pleomorphic lipoblasts (arrow)”.

On microscopic examination, the lesion is composed of two to three cell layers thick, benign mammary duct epithelium lining the slit-like spaces, and a cellular spindle cell stroma (Fig. 4a). The cellular mesenchymal stromal elements protrude into cyst-like spaces in a leaf-like configuration (Fig. 4b). Sarcomatous appearing stromal cells were bizarre spindle cells with large, crowded, pleomorphic nuclei. There was a slight mitotic activity (2 mitoses/10 HPF). No necrosis, hemorrhage or lymphovascular invasion were found. Stroma, described above, constituted a narrow zone, and continued with adipose tissue. Adipose tissue contained a significant number of bizarre cells; with a large cytoplasm and a lobulated nucleus. These cells were evaluated as “pleomorphic lipoblasts” (Fig. 5a–b), and the adipose tissue was diagnosed as pleomorphic liposarcoma component. Immunohistochemistry revealed that liposarcomatous elements were positive for S-100, and vimentin. The stromal cells were positive for vimentin, but negative for s 100, desmin and actin. The case was diagnosed as “Borderline Philloides Tumor with a Pleomorphic Liposarcoma Component”

## 3 Discussion

The majority of phyllodes tumors (up to 60%) are benign and are composed of cellular stroma formed from spindled cells, which are somewhat plump and show mild cytologic atypia and few mitoses (less than 5 per 10 high-power fields). Occasional bizarre pleomorphic stromal cells may be seen, but should not lead one to make the diagnosis of malignant phyllodes tumour in the absence of other features [5].

A variety of associated malignancies can arise from PTs, with its dual population of cells [8]. The stromal cells can demonstrate sarcomatous differentiation while the epithelial component can become malignant with DCIS/LCIS or invasive carcinoma [9]. Heterologous sarcomatous elements; such as liposarcoma, chondrosarcoma, or, osteosarcoma; may be seen in the stroma of PTs in 20% of the cases [5,10]. Several cases with coexistance of epithelial and sarcomatous component were also reported [8,9,11].

Histological grading systems for PTs is 3 tiered; “benign”, “low-grade malignant (borderline)”, or “high-grade malignant” according to the histopathologic features mainly based on the mitotic activity, type of margin, stromal overgrowth, and cellular pleomorphism [1,6].

Low-grade malignant or borderline PT, may have a microscopically circumscribed or invasive border, an average of two to five mitoses per 10 HPF, and moderate stromal cellularity that is often heterogeneously distributed in the midst of hypocellular. Infrequent instances of cartilaginous, osseous, and lipomatous metaplasia have been encountered in borderline PTs [1]. Low grade PT, has a potential for local recurrence, but it is very unlikely to metastasize. A low-grade PT is a tumour with low malignant potential [4].

*High-grade or malignant PT) has an infiltrating* or pushing margin, stromal overgrowth, moderate to severe nuclear atypia. Cut off value for mitosis, differs from 3 to 5 per 10 HPF according to different authors, respectively [1,4]. A high-grade PT is a tumour with high malignant potential.

According to the grading criteria of PT, our case suits in borderline category with a mitotic count of 2/10 HPF. This is similar to the case reported by L. Uriev et al., which has a liposarcomatous component and mitotic count of 2–3 per 10 high power fields [12]. Heterologous sarcomatous elements usually accompany high grade PTs; while Powell and Rosen, described 7 low-grade malignant PTs, with a mixoliposarcoma component [2]. A recent consensus report pointed the presence of a malignant heterologous component places the tumour into the malignant category regardless of other histological features [14,15].

Malignant PTs must be distinguished from metaplastic carcinoma and, rarely, primary sarcomas of the breast. Pure sarcomas (liposarcoma, chondrosarcoma, etc.) without relationship to a mammary PT are extremely rare; one needs to exclude the possibility of a PT [16].

Malignant epithelial component in metaplastic carcinomas, tends to merge with the spindle cell element, whereas in PT, the epithelial component is benign and remains discrete from the spindle cell component. A panel of cytokeratin markers (including high-molecular-weight cytokeratins) and p63 should be assessed immunohistochemically and can be particularly useful in highlighting the malignant epithelial spindle cells in metaplastic carcinoma. CD34 may also be of value; it is often expressed by the stroma of PTs but is not seen in spindle cell metaplastic carcinoma or fibromatosis. Our case had a negative pancytokeratin and CD 34 staining.

A variety of other immunohistochemical markers, such as Ki-67 p53, VEGF, EGFR, CD10; flow cytometry analysis of ploidy and S-phase, cytogenetic studies and quantitative measures of stromal cellularity, stromal-to-epithelial ratio, mitotic rate, stromal overgrowth, and mean nuclear diameter were studied, in order to distinguish cellular fibroadenomas from benign phyllodes tumors and subclassify benign, borderline, and malignant phyllodes tumors. Although Ridgway et al. reported K -67 as a significant indicator, most of these ancillary techniques were however, not proved to be of great practical help for diagnostic use [1,4,17].

When a phyllodes tumour recurs, it can do so as pure sarcoma, without epithelial elements; therefore, a history of a previous phyllodes tumour should be sought in such cases. Approximately 30% of PTs, develop recurrences, within 2–3 years after the diagnosis. Recurrences may show more aggressive histologic features such as increased cellularity, significant nuclear atypia, and increased mitotic activity [4].

Metastases are hematogenous (lung, bone, heart, liver, etc.) and occur in less than 10% of the cases, within 2 years of the initial surgery. Lymph node metastases are less than 1% of high-grade PTs [4].

### 3.1 Therapy

There is a paucity of evidence regarding surgical and adjuvant therapy. Although the literature often refers to a margin width of at least 10 mm [8]. A clear evidence to support this approach is lacking. Therefore, an ideal margin width remains to be determined, and may need to be considered in relation to factors such as tumour size and cosmesis [14]. The fascial site was the closest margin in our case (3 mms). The lesion was free of margin in 2 cms of peripheral breast tissue. No reexcision was performed. The role of adjuvant radiation therapy in borderline and malignant tumors remains to be defined. Routine axillary dissection is not recommended [14].

Following definitive surgery, the patient underwent a reconstructive operation (subcutaneous mastectomy with breast implant replacement). There were not any complications after any of the surgeries. The case was discussed at the local multi-disciplinary meeting and the later treatments were recommended to follow European Sarcoma guidelines, by the oncology department [18]. Unfortunately the follow up was not possible because patient did not come to control appointments.

Diagnostic algorithms and usual morphological radiologic features sometimes may not be so useful on daily life practice. BIRADS is mostly useful for categorisation of breast masses and for the common language between the radiologists and surgeons. However, BIRADS may not be such useful for some exceptional lesions. Fat containing lesions such as liposarcoma can be misdiagnosed as fibroadenolipoma on mammograms and ultrasounds. Also phillodes tumour’s sonographic features are similar to fibroadenoma as they can have smooth contours. Any suspicious lesion, detected on breast imaging, should be biopsied. Sonography guided biopsy is a safe and easy method. US should be followed by CT or MRI in those similar suspected lesions.

Histopathology results should be discussed carefully and total excision must be performed if there is any discordance with the radiologic features.

The diagnosis of a phyllodes tumour should be considered in rapidly growing cases, particularly over the age of 35 years. All breast lumps should be examined by a multidisciplinary -clinical, radiological, and pathological- approach.

## Conflicts of interest

There is no conflict of interest.

## Sources of funding

There are no sources of funding for our research.

## Ethical approval

Ethical approval has been exempted by our institution.

## Consent

We don’t have signed consent from the patient, guardian or family. Exhaustive attempts have been made to contact the family and the paper has been sufficiently anonymised not to cause harm to the patient or their family.

Abut Kebudi, MD, PROF, FACS Head of the Medical Team.

## Author contribution

Yalçın Polat study concept and design, writing the paper.

Gözde Arslan data collection.

Abud Kebudi, data collection.

Duygu Düşmez Apa study concept and design, writing the paper.

## Registration of research studies

NA.

## Guarantor

DR. Yalçın Polat AND Dr. Duygu Düşmez Apa.

## Provenance and peer review

Not commissioned, externally peer reviewed.
